# The intricate relationship between microtubules and their associated motor proteins during axon growth and maintenance

**DOI:** 10.1186/1749-8104-8-17

**Published:** 2013-09-08

**Authors:** Andreas Prokop

**Affiliations:** 1 Faculty of Life Sciences, Michael Smith Building, Oxford Road, Manchester M13 9PT, UK

**Keywords:** Axons, Growth cones, Cytoskeleton, Microtubules, Kinesins, Dynein, Transport, Brain disorders, Drosophila

## Abstract

The hallmarks of neurons are their slender axons which represent the longest cellular processes of animals and which act as the cables that electrically wire the brain, and the brain to the body. Axons extend along reproducible paths during development and regeneration, and they have to be maintained for the lifetime of an organism. Both axon extension and maintenance essentially depend on the microtubule (MT) cytoskeleton. For this, MTs organize into parallel bundles that are established through extension at the leading axon tips within growth cones, and these bundles then form the architectural backbones, as well as the highways for axonal transport essential for supply and intracellular communication. Axon transport over these enormous distances takes days or even weeks and is a substantial logistical challenge. It is performed by kinesins and dynein/dynactin, which are molecular motors that form close functional links to the MTs they walk along. The intricate machinery which regulates MT dynamics, axonal transport and the motors is essential for nervous system development and function, and its investigation has huge potential to bring urgently required progress in understanding the causes of many developmental and degenerative brain disorders. During the last years new explanations for the highly specific properties of axonal MTs and for their close functional links to motor proteins have emerged, and it has become increasingly clear that motors play active roles also in regulating axonal MT networks. Here, I will provide an overview of these new developments.

## Review

### Introduction

**Figure 1:**
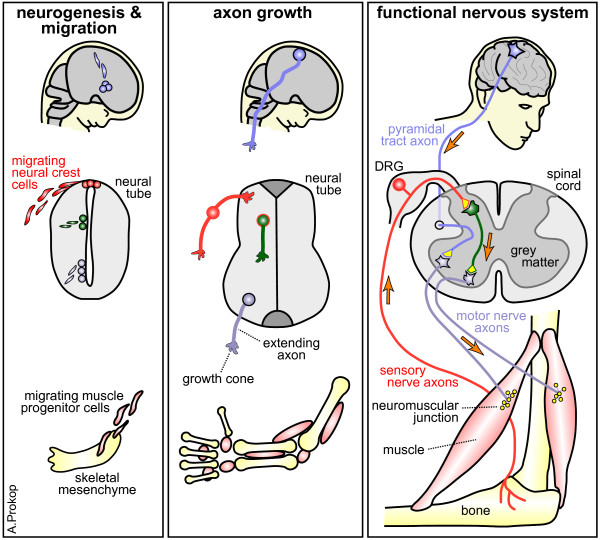
**The growth and function of axons.** The process of neurogenesis provides pools of neurons that migrate into their appropriate positions (left), that extend axons along reproducible paths (middle), and that are eventually wired up into the synaptic circuits which convey information across the nervous system, thus coordinating behavior (orange arrows: action potentials; yellow: presynaptic sides).

Axons are the longest cellular processes produced by animals. They conduct action potentials away from the neuronal cell body to pass them on to other cells at synapses (Figure 1) [1]. They are slender nerve cell extensions that electrically wire up the brain and establish the information highways that essentially underpin nervous system function. Axons can be up to a meter long in humans, especially those axons that are bundled into nerves of the body or into nerve tracts in the CNS (Figure 1). These remarkable cellular structures need to be fabricated during development and maintained for the lifetime of an animal, which in humans is for decades. This long-lasting maintenance is an enormous logistical challenge where a tiny neuronal cell body sustains a cell compartment that is up to weeks away in terms of cargo transport duration - in relative dimensions comparable to the communication and supply lines which Alexander the Great or Hannibal faced when sustaining their war campaigns far away from their homelands [2]. In architectural terms, axons form not just a stable, cemented structure, but mature axons have the principal ability to undergo plastic reorganization underpinning learning and memory, and may re-grow after injury in order to regain lost control over body movement and behavioral abilities [3,4].

Clearly, axons are masterpieces of biology and their study has fascinated neurobiologists since they were first described in the second half of the 19th century [1]. Their study is important and will have important implications when considering the increasing social burden of brain disorders [5]. For example, axons undergo incremental loss during ageing, reaching 50% loss at 80 years [6], and understanding the reasons underlying this loss will address age-related brain disorders. Axons die back in many neurodegenerative diseases, and we will learn a lot from understanding whether this is cause or consequence of neuronal decay [7-9]. Failed re-growth of axons after spinal cord injury is an important cause of sustained paralysis, and learning ways to stimulate axonal re-growth and regeneration clearly addresses issues of life quality and economic burden [4].

To address such issues, it is pivotal to gain a fundamental understanding of the cell biology of neurons, in particular the role and regulation of axonal microtubules (MTs) which are the centerpieces of axons and have long been recognized as displaying unique features when compared to non-neuronal cells [10]. Important progress has been made over the last years in understanding these features, and it has become increasingly clear that there are close links between MTs, the process of axonal transport, and the motors which perform this function. In this review, I will give a brief overview of the role and regulation of MTs in axons, describe their links to axonal transport and their intricate relationship with MT-associated motor proteins, the functions of which are not only transport-related.

### MTs in axons display specific properties and essentially drive axon growth

**Figure 2:**
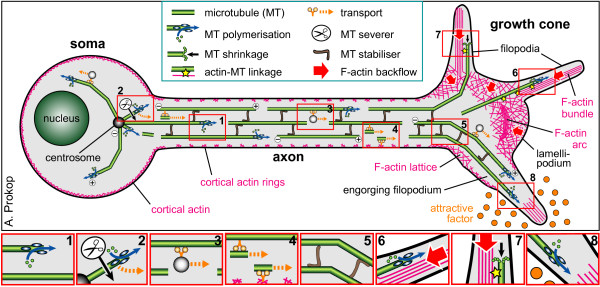
**Axon structure and key mechanisms of axon growth.** The image shows a neuron which extends an axon into a zone of an attractive factor. Parallel bundles of MTs fill the axon and splay in the growth cone (plus end-out polarity indicated for some MTs by encircled + and - signs). F-actin networks are prominent in growth cones, and scarce but highly organized into evenly spaced rings in axons. Close-ups illustrate the following molecular mechanisms contributing to axon growth: (1) MT plus end-associated factors (for example, EB1, CLASP, type 13 kinesins, XMAP215; blue ellipses) regulate elongation and shrinkage of MTs (blue arrows); (3) molecular motors (orange Y structures) mediate cargo transport; (2,4) the MT severing protein katanin (scissors) generates MT fragments which are moved anterogradely through MT-sliding; (5) proteins that bind along MTs (for example, tau, MAP1B; brown L's) protect MTs from depolymerization or severing factors and organize MTs by cross-linking them and regulating their spacing; (6–8) F-actin networks influence MT behaviors through antagonizing MT advance into lamellipodia and filopodia via retrograde flow (red arrows), through forming structures that can be supportive (for example, radial bundles) or inhibitory (for example, transverse arcs), through MT cross-linkage (yellow stars), through contractile activity (not shown), or through F-actin clearance from protrusions (shown in 8). For detailed information see [3,23].

**Figure 3:**
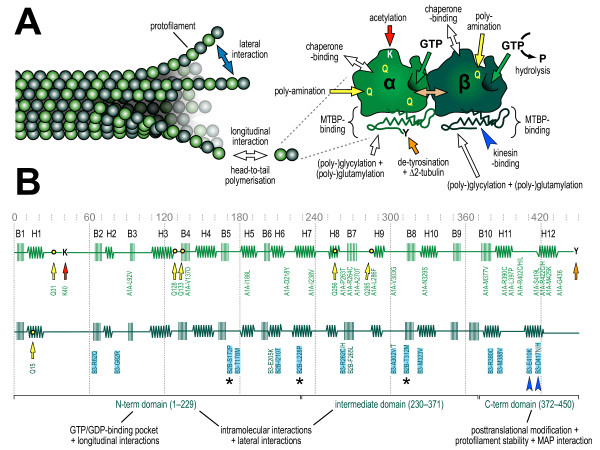
**Structural aspects of tubulins and microtubules (MTs). (A)** MTs grow through head-to-tail polymerization of α-/ß-tubulin heterodimers into protofilaments that arrange into hollow tubes through lateral interactions. The α-and ß-tubulin monomers need to be folded properly assisted by chaperones, they heterodimerize through longitudinal interactions (peach arrow), they bind GTP (of which GTP on ß-tubulin tends to undergo hydrolysis to GDP) and undergo a number of posttranslational modifications, including de-tyrosination (often followed by de-glutamylation resulting in ∆2-tubulin), acetylation, poly-amination, (poly-)glycylation and (poly-)glutamylation [11]. MTBPs primarily interact with the C-terminus of tubulins which sticks out from the MT surface. **(B)** The secondary structure of α- and ß-tubulin (color-coded as in A) showing the positions of ß-sheets (B1-10) and α-helices (H1-12; image modified from [24]); the borders and principal functions of the N-terminal, intermediate and C-terminal domains of ß-tubulin are indicated at the bottom and arrows indicate examples of posttranslational modification sites [11,21]. Positions of known dominant-negative mutations are shown below the two secondary structures [25]. Those mutations tested by Niwa and colleagues are highlighted in light blue, asterisks indicate those mutations that impair tubulin incorporation into MTs and blue arrowheads indicate the two charge-changing mutations in H12 [26].

MTs form the structural backbones of axons (Figure 2). They are stiff hollow tubes typically composed of 13 parallel protofilaments. These protofilaments are polar filamentous polymers of α-/ß-tubulin heterodimers which frequently display posttranslational modifications including (poly-)glycylation and (poly-)glutamylation on the C-termini of both α- and ß-tubulin, as well as α-tubulin-specific acetylation and detyrosination (often followed by de-glutamylation, resulting in ∆2-tubulin) [11,12] (Figure 3). In contrast to MTs in non-neuronal cells, axonal MTs display a number of special features. First, axonal MTs range in length from just a few μm to many tenths of μm and they are arranged into evenly spaced prominent parallel bundles where most MTs point with their plus ends towards the axon tip [13,14]. The mechanisms that bundle MTs are little understood, but are believed to involve MT-binding proteins (MTBPs) such as MAP1B or tau (Figure 2) [15,16]. Second, the nucleation of axonal MTs does not require the centrosome in the cell body (Figure 2), but can occur from diffuse sites in axons [17,18]. Third, ß-tubulin-associated guanosine triphosphate (GTP) is usually hydrolyzed to guanosine diphosphate (GDP) once incorporated into MTs (Figure 3), whereas axonal MTs maintain a high level of GTP-tubulin [19] with likely implications for their stability [20]. Fourth, sub-fractions of axonal MTs become polyaminated by transglutaminases (Figure 3) which renders these MTs stable to cold- and calcium-treatment [21]. Finally, axonal MTs of neurons are usually interwoven with intermediate filaments which are highly abundant in axons, are known to regulate the specific diameters of different axon classes, and have been associated with neurodegenerative processes [22].

Axon growth is essentially implemented through the extension of axonal MT bundles, and net positive polymerization of MTs is expected to contribute essentially to this growth (Figure 2). This notion is best illustrated by mutations in tubulin genes or tubulin chaperones such as tubulin folding cofactor E (Tbce) which negatively impact on MT polymerization, and which have been linked to neuro-developmental diseases including impaired axon growth [25,27]. There is some evidence for elongation within the axon shaft, which involves anterograde propulsion of long MTs and slow incremental forward flow of the entire MT mass toward the leading growth cone [28,29]. Such a finding is in principle consistent with the idea of *de novo* polymerization at disperse nucleation sites or at the plus ends of MTs along axon shafts [10,17]. Certainly this model needs further confirmation, but it could provide interesting explanations for how the tips of axonal MT bundles can push in growth cones during the process of protrusion, engorgement and consolidation which implement axon extension [23,30,31].

MTs polymerize (grow) and depolymerize (shrink) primarily at their plus ends, and these processes, as well as the directionality of MT extensions, are essentially regulated through tip interacting protein (+TIPs). +TIPs localize to MT plus ends primarily through interaction with end-binding proteins (EBs) which can directly bind to those MT plus ends that undergo polymerisation [3,23,32]. This said, β3-tubulin can also directly interact with membrane receptors during axon growth and guidance [33]. MTBPs and +TIPs regulate further aspects of MT dynamics, such as stability and cross-linkage into bundles and MT cross-talk with F-actin, the cell cortex, organelles and transported cargo (Figure 2), and some of these activities have been shown to contribute to axon growth [3,23]. However, as argued recently [23], knowing these single molecular or subcellular mechanisms and their principal impacts on axon growth, is still far from understanding axon growth. We need to acknowledge that the various molecular mechanisms of different MTBP classes (as well as of actin- and intermediate filament-regulating proteins) integrate into one common and complex cytoskeletal machine. Taking out a single component does not bring the machinery to a halt, but may significantly change the way it works and cause phenotypes that are difficult to interpret. Therefore, we need to find strategies to decipher this machinery across its various components and to understand their functional interfaces.

### Axonal microtubules provide the highways for motor-driven cargo transport

As stated above, communication of a neuronal cell body with distant segments of its axon poses a serious logistical challenge and involves long-distance axonal transport of a wide range of different cargoes including lipids, different protein classes (usually transported via cargo vesicles), organelles as large as mitochondria, but also mRNAs [34,35]. This transport occurs along the axonal MT bundles and is driven by dynein/dynactin and kinesin motor protein dimers/complexes which use pairs of motor domains to step along MTs in a ‘hand-over-hand’ mode at a speed of ≤ 1 μm/s (Additional file 1: Table S1) [34,36]. These molecular motors require ATP as an essential energy source. The major producers of ATP in cells are the mitochondria, but molecular motors will only occasionally encounter mitochondria on their axonal journey. A recent report suggests that this logistical problem is solved via a system of ‘on-board’ ATP provision in form of the enzyme GAPDH (glyceraldehyde 3-phosphate dehydrogenase), which localizes to the cargo vesicles and contributes to the break-down of glucose from the neuronal cytosol [37].

Retrograde transport (that is, towards the cell body) is mediated by cytoplasmic dynein/dynactin which is a fairly large and multi-component protein complex adaptable to all kinds of cargos and functional tasks (Additional file 1: Table S1) [36,38]. Anterograde transport (that is, away from the cell body) is driven by kinesin motor proteins. Forty-five different kinesins grouped into 14 families are known in mammals, of which hetero-oligomeric type 1 and 2 as well as homodimeric type 3 kinesins are the most prevalent mediators of anterograde transport in axons (Additional file 1: Table S1) [34]. The regulation of transport speed and direction is only partly understood and involves guidance through neuronal architecture, signaling mechanisms, distinct qualities and modifications of the various motors, linkers and otherwise associated proteins, posttranslational modifications of MTs and cargo, as well as complex interactions of motors with other motors and MTBPs (Additional file 2). Understanding this transport machinery is of importance as emphasized by the many links that mutations in the various kinesin and dynein/dynactin genes have to developmental and neurodegenerative brain disorders [8,34,39-41].

### Charge-changing mutations in tubulins translate into roadblocks for migrating kinesins

Also mutations in tubulin genes have been linked to human brain disorders (Figure 3) [25]. Mammalian genomes encode six classes of ß-tubulins (with TUBB1, 2 and 3 being most abundant in the brain), and four classes of α-tubulins. Given the high degree of sequence conservation between tubulins, they are likely to be able to functionally replace each other, at least partially. It seems therefore logical that most disease-linked tubulin mutations discovered to date are of dominant-negative nature, that is, mutant tubulins need to be incorporated into MTs or their polymerization machinery to alter MT functions or dynamics and impact on cellular behaviors. Some of these mutations are known or speculated to affect MT polymerization or stability interfering with protein folding and/or chaperone interactions, α-/ß-heterodimerization, head-to-tail polymerization of dimers, the GTP-binding ability (important for MT dynamics), or the ability to establish lateral bonds between protofilaments (Figure 3) [25]. Other tubulin mutations are far less understood, but can be expected to alter interactions with different classes of MTBPs, and studies in non-neuronal cells or yeast suggested interference of some mutations with molecular motors [25,42].

Clear evidence that certain tubulin mutations affect the MT interaction with kinesins in axons has now been provided by the studies of Niwa and collaborators [26]. They transfected cultured hippocampal neurons with 14 different mutant ß-tubulins (highlighted in Figure 3) and found that two of them, TUBB3^E410K^ and TUBB3^D417H^, suppressed anterograde axonal cargo transport as well as the ability of type 1, 3 and 4 kinesins (that is, major players in axonal transport) to move to axon tips. Of these, the type 4 kinesin KIF21A is of particular interest: it acts as a genuine axonal transporter [43] and its mutations have been linked to type 1 congenital fibrosis of the extraocular muscles (CFEOM), a pathology that affects nerve growth to certain eye muscles [44]. The TUBB3^E410K^ and TUBB3^D417H^ mutations cause a very similar pathology (type 3 CFEOM) [25], suggesting that these mutations functionally relate to KIF21A *in vivo*.

Both mutations are positioned in the H12 helix of ß-tubulin where they cause a negative-to-positive charge change (Figure 3, dark blue arrowheads), and a detailed structure-function analysis clearly demonstrated that the negative charge of H12 is crucial to properly support kinesin attachment to MTs [26]. Introduction of the E410K or D417H mutations into TUBB2 or TUBB5 caused similar transport defects, as long as these mutant tubulins were strongly expressed in neurons. However, if these mutant tubulins were prevented from assembling into MTs (by inserting a second site mutation which structurally prevents this tubulin from incorporation), their dominant-negative impact on MTs was abolished. A 10% incorporation rate of mutant tubulins into MTs was estimated to be sufficient to interfere with kinesin transport to a degree that becomes disease-relevant [26], and only brain expression of TUBB3 is high enough to achieve this degree of incorporation.

In conclusion, this work convincingly explains a molecular link between tubulins and kinesins and how their molecular properties and interactions can cause the CFEOM disorder as a common systemic outcome. Clearly, it links molecular mechanisms to cellular effects, and it nicely combines the two areas of neuronal tubulin and kinesin research in a way that expands our understanding of neuronal cytoskeletal machinery.

### MT motor proteins contribute to axon growth through their transport function

Transfection of neurons in the embryonic mouse brain with TUBB3^E410K^ and TUBB3^D417H^ caused reduced axon growth, and the same phenotype was observed when knocking down the type 3 kinesin KIF1B [26], suggesting that functional interactions of kinesins with MTs promote axon development. Similar observations were made for other axonal transport motors, in particular with loss of function of kinesin-1 and dynein/dynactin, which likewise caused reduced axon growth [45-47]. Furthermore, the loss of kinesin-3 or of dynein/dynactin components in *Drosophila* causes axonal aberrations, in particular of the branching and differentiation of their synaptic terminals [48-51]. This poses the important question of how the loss of motor activity affects axon growth and morphogenesis. The answer to this question is not trivial.

One obvious mechanism through which motors support axon growth is through cargo transport [52]. Thus, any growth process is in need of building materials, such as lipids, structural proteins, signaling pathway components and cell organelles which can not all be produced at the growth cone. At least part of them, or the mRNAs encoding them, have to be delivered through the axonal supply line from the cell body. This requirement is nicely illustrated by a classical experiment in which growing axons of frog retinal ganglion cells were cut off from their cell bodies *in vivo*; the longer the axon fragment attached to the growth cones was, the longer these growth cones survived and continued to execute their proper growth program (up to three hours), suggesting that the attached axon fragments provided a function- and life-sustaining supply pool [53]. However, aberrations of axonal transport might cause axonal growth phenotypes not only through depletion of supplies, but could trigger other pathomechanisms which secondarily impact on axon morphogenesis and differentiation. For example, a hypomorphic allele of the *Drosophila Unc4* gene (kinesin-3) was recently reported to show overgrowth at the neuromuscular junction [50]. Very similar phenotypes are observed when inducing oxidative stress [54], suggesting a potential mechanism for this phenotype that can easily be tested in the fly system (Additional file 3).

### MT motor proteins contribute to axon growth through direct regulation of MT networks

Also MTs are amongst the cargos transported in axons. Originally this has been considered to be slow axonal transport (0.1 to 3 mm/day) performed by KIF5/kinesin light chain (KLC) activity [52]. This view has been disputed after different experimental procedures revealed rapid bi-directional movement of short MT fragments of ≤ 10 μm length [55,56]. Dynein/dynactin has been shown to mediate MT fragment movement through ‘MT sliding’, that is, by anchoring to long MTs or F-actin and driving MT fragments anterogradely by walking towards their minus ends (Figure 1, inset 4), and this function seems to contribute to axon growth [56-58]. It has been suggested that transported MT fragments are very stable (likely through polyamination) and could therefore promote axon growth by serving as powerful nucleation sites for new MTs along axons [10]. In agreement with this idea, blocking polyamination severely inhibits axon growth, although the underlying mechanisms have so far not been resolved [21].

This example of MT fragment transport illustrates that molecular motors not only depend on MTs for their transport functions but, *vice versa*, they also influence MT dynamics and behaviors, that is, they regulate the construction of the highways they will later travel on. Further examples for this have come to light. For example, type 5 (KIF11), type 6 (KIF23) and type 12 (KIF15) kinesins are best known for their MT sliding roles during mitosis. In developing axons these kinesins are growth inhibiting and seem to regulate axon growth, branching and growth cone turning through antagonizing dynein-mediated MT fragment transport and through regulating the extension of MTs in growth cones [59-62]. Furthermore, dynein and its associated factor Lis1 (Additional file 1: Table S1) have been suggested to antagonize inward-directed forces imposed by retrograde actin flow, thus helping MTs to extend into the growth cone periphery and promote axon growth [63-65]. The dynactin complex component p150 Glued plays direct roles in MT catastrophe regulation in axons [66]. Type 13 kinesins, such as KIF2A, are minus end-directed motors (not contributing to transport) which can be recruited to MT plus ends and actively disassemble them, and this function inhibits collateral branch formation of axons *in vivo*[67].

Also type 1 kinesins have recently been implicated in MT regulation during axon growth. Thus, the *Drosophila* KIF5 homolog kinesin heavy chain (KHC) was shown to drive axon initiation and transiently maintain axon growth (though to a lesser degree), even if MT polymerization or axonal transport were blocked [68]. This function did not require the transport-relevant KLC, and the authors proposed that KHC can cross-link MTs via its N-terminal motor domains and C-terminal MT-binding sequences. A similar mechanism was described for fly KHC as well as its frog homolog KIF5 in non-neuronal cells [69]. Notably, KIF5 is one of the first factors accumulating at axon initiation sites in mammalian neurons [70] and, if the MT sliding mechanism applies, it could similarly help MTs to push and initiate newly forming axons in mammals. Since KIF5 is also strongly enriched in growth cones [26,70], it might as well play a role in growth cone extension and/or turning, in parallel to the roles of KIF11 mentioned above [59].

In conclusion, molecular motors perform functions that clearly reach beyond mere transport roles suggesting that they display prominent mutual dependencies with MT network regulation.

### The roles of transport, MTs and MT motor proteins in axon maintenance

Apart from their developmental roles, molecular motors certainly play major roles also in mature neurons. Thus, they continue to sustain the supply and communication lines that maintain neuronal physiology, and the transport of synaptic components, and of mitochondria to meet the high energy demand of synapses [71]. Therefore, MT motors are essential for neuronal longevity, as is also indicated by the numerous mutations in kinesin and dynein/dynactin genes which link to neuropathies and neurodegeneration [8,34,39-41], as well as by the fact that the H12 loop mutations of TUBB3 link to neuropathies [25]. Intriguingly, axons in the ageing or demented brain frequently display swellings or diverticula, which are areas of looped and criss-crossed MTs posing potential transport traps [9,72,73]. Thus, in addition to mutations that affect the molecular motors or their binding to MTs, the turning of straight axonal MT tracks into spaghetti junctions where cargos get blocked through steric hindrance seems to be an important mechanism of interference with axonal transport, and this effect has been reproduced through mathematical modeling [74].

Since the appearance of diverticula seems to increase with ageing, research needs to be directed to the investigation of the underlying causes, that is, the study of mechanisms that maintain parallel MT bundles in healthy neurons. So far, a number of potential mechanisms were identified. Thus, structural MAPs (for example, tau and MAP1B) have long been suggested as playing an important part in maintaining MT bundles [15]. The level of cold-stable polyaminated tubulin increases in older neurons, suggesting that MT stability plays an important part [21]. Mutations affecting the MT-severing spastins cause axon swellings and transport defects in mouse models and humans [75,76]. Intermediate filaments are closely interwoven with MTs and might play roles in their ordered maintenance [22]. However, MT bundles in mature axons are not only highly stabilized structures, but they are likely to undergo constant renewal through MT polymerization [77], as is also indicated by the linkage of neuropathies to the misregulation of stathmins, powerful regulators of MT polymerization [76-81] or the tubulin chaperone Tbce [27,82,83]. This suggests that some mechanisms acting in development might be maintained at adult stages, as recently proposed for the actin-MT linking spectraplakins. Spectraplakins guide MTs along actin structures (potentially cortical actin; Figure 2) and lay them out into parallel bundles during axon growth, and their loss causes MT disorganization [84,85]. Analogously, loss of the spectraplakin dystonin causes MT disorganization at postnatal stages and this correlates with sensory neuropathy [23,86].

Therefore, it is tempting to speculate that developmental roles of MT motors in MT organisation [45,63-66] might likewise apply at adult stages. Support for this notion is provided by KIF11 which negatively regulates axon growth during development, but also during axonal regeneration after injury in the adult brain [87]. As a further example, loss of dynein/dynactin from *Drosophila* neurons induces an increase in wrongly oriented minus end-out MTs in axons [88], and this is almost certain to cause transport aberrations. To explain this phenomenon, an appealing model has been put forward suggesting that dynein/dynactin might remove wrongly oriented MTs through retrograde MT transport, instead of chopping them up, like Hydra's head that would sprout new ones in their place, by using generated MT fragments as nucleation sites [56]. These few examples may only be the tip of the iceberg and more MT-regulating roles of molecular motors contributing to the cellular processes underlying axon maintenance might well be discovered, but not necessarily roles and mechanisms that might be expected at this stage.

## Conclusions

There is an intricate relationship between MTs, the mechanisms that drive their nucleation, (de-)polymerization, cross-linkage and modification, and the motors that use them for transport but also influence them in their regulation. Given the disease relevance of this regulatory machinery it is pivotal that we gain a better understanding of its workings. Yet, this task is challenging; it requires multidisciplinary approaches and a stronger integration of the various lines of research in the areas of tubulin regulation and MT dynamics as well as motors and transport. Importantly, we need neuronal systems in which studies of the various molecular components and functional contributions can be integrated, and this cries out for the use of simpler invertebrate model organisms such as *Caenorhabditis elegans* or *Drosophila*, which are well known for their power to work out fundamental concepts and mechanisms that can then be applied in higher animals or studies of human disease (Additional file 3) [23,89]. One essential factors that drives such research in invertebrate models is their low genetic redundancy (Additional file 1: Table S1) which facilitates functional removal of whole gene classes, alone or even in combination [90,91]. Notably, cellular models for nervous system development, injury and disorders in which the axon-relevant roles of tubulin, MT regulators and motors can be studied are already well established in *Drosophila*[92-94], and various examples cited in this review come from fly studies. Furthermore, attempts have already been made to simulate *Drosophila* axons in mathematical models [95,96], thus paving the way towards experimental assembly lines that can integrate molecular mechanisms with genetic analyses of their cellular functions and develop them into mechanistic models that will eventually be able to predict the outcome of single mutations and deliver explanations for how they cumulate in disease phenotypes.

Naturally, the fly model has certain limitations, such as the far shorter length and life span of axons, but these differences might also provide opportunities. For example, the plekstrin homology domain-containing type 3 kinesins KIF1A and KIF1Bß and their fly homolog Unc-104 (Additional file 1: Table S1) have been implicated in transport of synaptic vesicles [52]. Whereas deletion of KIF1A or KIF1Bß function causes reduced axon growth in the mouse brain [26,97], loss of Unc-104 in fly does not, although it shows deficits in the final morphology of synaptic terminals [50,51]. This might indicate functional deviation but, more likely, it reflects cellular differences where aberrant cargo transport has a greater impact in the much longer mouse axons. These possibilities are easy to test via inter-species rescue experiments and might therefore offer exciting experimental means to distinguish between roles of motors in cargo transport and their roles in MT regulation.

## Abbreviations

EB: End binding protein; GADPH: Glyceraldehyde 3-phosphate dehydrogenase; KLC: Kinesin light chain; KHC: Kinesin heavy chain; MT: Microtubules; MTBP: Microtubule-binding protein; +TIP: Tip-interacting protein.

## Competing interests

The authors declare that they have no competing interests.

## Supplementary Material

Additional file 1: Table S1 Microtubule (MT)-associated motor proteins with roles in axons [61,62,98-104]. Click here for file

Additional file 2 **Regulation of axonal transport **[10,12,19,34,36,71,97,98,105,106,107,108,109,110,111,112,113,114,115,116,117,118]**.** Click here for file

Additional file 3 **Using the fruit fly *Drosophila *to study the cytoskeleton during axon growth **[23,85,89,91,119,120,121,122]**.** Click here for file
