# Supplementary material for: The intricate relationship between microtubules and their associated motor proteins during axon growth and maintenance
Source: Neural Dev. 2013 Sep 8;8:17. doi: 10.1186/1749-8104-8-17 (PMC3846809; doi:10.1186/1749-8104-8-17)
Supplement: Additional file 1: Table S1 — Microtubule (MT)-associated motor proteins with roles in axons [61,62,98-104]. [file 1749-8104-8-17-S1.doc]

| **Type** | **Structure** | **All**  **in mouse** | **Fly genes** | **Expression of fly kinesins** | **Assigned mouse homolog** |
| --- | --- | --- | --- | --- | --- |
| **1**  N-Kifs | 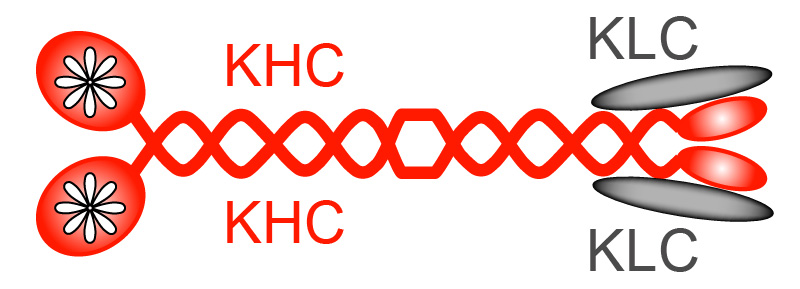 | **3** - [Kif5A](http://www.informatics.jax.org/marker/MGI:109564), [5B](http://www.informatics.jax.org/marker/MGI:1098268), [5C](http://www.informatics.jax.org/marker/MGI:1098269) | - [KHC](http://flybase.org/reports/FBgn0001308.html) (motor) | all stages; strong in **NS** | Kif5A, 5B, 5C |
|  |  | **4** - [Klc1](http://www.informatics.jax.org/marker/MGI:107978), [2](http://www.informatics.jax.org/marker/MGI:107953), [3](http://www.informatics.jax.org/marker/MGI:1277971), [4](http://www.informatics.jax.org/marker/MGI:1922014) | - [Klc](http://flybase.org/reports/FBgn0010235.html) (light chain) | all stages; strong in **NS** | Klc1, 2, 4 |
|  |  | **2** - [Nae1](http://www.informatics.jax.org/marker/MGI:2384561), [Appbp2](http://www.informatics.jax.org/marker/MGI:1914134) | - [Pat1](http://flybase.org/reports/FBgn0029878.html) (cargo linker) | all stages; strong in **NS** | Appbp2 |
| **2**  N-Kifs | 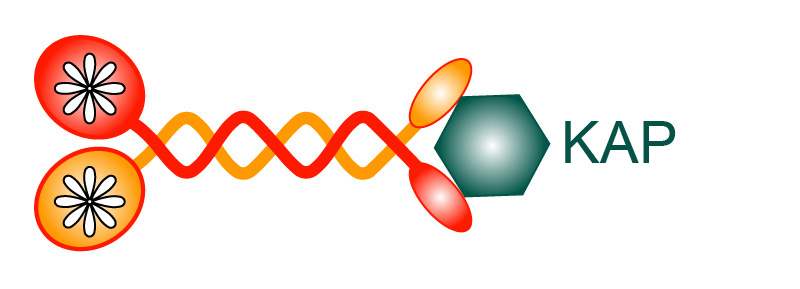 | **4** - [Kif3A](http://www.informatics.jax.org/marker/MGI:107689), [3B](http://www.informatics.jax.org/marker/MGI:107688), [3C](http://www.informatics.jax.org/marker/MGI:107979), [17](http://www.informatics.jax.org/marker/MGI:1098229) | - [Klp64D](http://flybase.org/reports/FBgn0004380.html)/ Kif3A | all stages; strong in **NS** | Kif3A, 3B, 3C, 17 |
|  |  |  | - [Klp68D](http://flybase.org/reports/FBgn0004381.html) | all stages; strong in **NS** |  |
|  |  |  | - [Kif3C](http://flybase.org/reports/FBgn0039925.html) | intermittent at all stages; moderate in **NS** |  |
|  |  | **1**(?) **-** [Kifap3](http://www.informatics.jax.org/marker/MGI:107566)/ Kap3 | - [Kap3](http://flybase.org/reports/FBgn0028421.html) (cargo linker) | all stages; strong in **NS** | (rat) Kifap3 |
| **3**  N-Kifs | 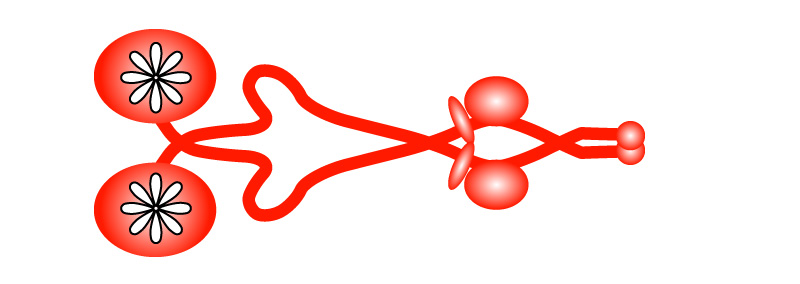 | **8** - [Kif1A](http://www.informatics.jax.org/marker/MGI:108391), [1B](http://www.informatics.jax.org/marker/MGI:108426), [1C](http://www.informatics.jax.org/marker/MGI:1098260), [13A](http://www.informatics.jax.org/marker/MGI:1098264), [13B](http://www.informatics.jax.org/marker/MGI:1098265), [14](http://www.informatics.jax.org/marker/MGI:1098226), , [16B](http://www.informatics.jax.org/marker/MGI:1098240), [16A](http://www.informatics.jax.org/marker/MGI:3045258)/ Stard9 | - [Unc-104](http://flybase.org/reports/FBgn0034155.html)/ Klp53D ([PH](http://e71.ensembl.org/Drosophila_melanogaster/Transcript/ProteinSummary?db=core;g=FBgn0034155;r=2R:12639004-12660002;t=FBtr0087079)) | all stages; strong in **NS**; some exons accessory gland-specific | Kif1A ([PH](http://www.ensembl.org/Mus_musculus/Transcript/ProteinSummary?db=core;g=ENSMUSG00000014602;r=1:93015456-93101865;t=ENSMUST00000171796)), 1Bß ([PH](http://www.ensembl.org/Mus_musculus/Transcript/ProteinSummary?db=core;g=ENSMUSG00000063077;r=4:149176319-149307693;t=ENSMUST00000055647)), 1Bα, 1C |
|  |  |  | - [Khc-73](http://flybase.org/cgi-bin/fbidq.html?FBgn0019968)/ Klp-73 ([CAP-Gly](http://e71.ensembl.org/Drosophila_melanogaster/Transcript/ProteinSummary?db=core;g=FBgn0019968;r=2R:11403283-11419759;t=FBtr0087357)) | all stages; strong in **NS** | Kif13A, 13B ([CAP-Gly](http://www.ensembl.org/Mus_musculus/Transcript/ProteinSummary?db=core;g=ENSMUSG00000060012;r=14:64652531-64806296;t=ENSMUST00000100473)) |
|  |  |  | - [Klp98A](http://flybase.org/reports/FBgn0004387.html) ([PX](http://www.ensembl.org/Drosophila_melanogaster/Transcript/ProteinSummary?db=core;g=FBgn0004387;r=3R:23373497-23384862;t=FBtr0085211)) | all stages; strong in **NS** | Kif16B ([PX](http://www.ensembl.org/Mus_musculus/Transcript/ProteinSummary?db=core;g=ENSMUSG00000038844;r=2:142617474-142901531;t=ENSMUST00000043589)) |
| **4**  N-Kifs | 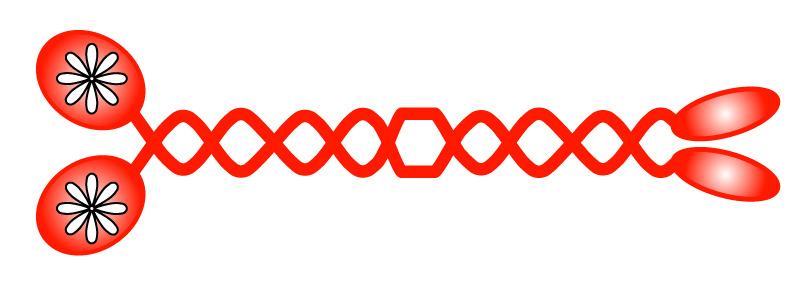 | **5** - [Kif4](http://www.informatics.jax.org/marker/MGI:108389), [7](http://www.informatics.jax.org/marker/MGI:1098239), [21A](http://www.informatics.jax.org/marker/MGI:109188), [21B](http://www.informatics.jax.org/marker/MGI:109234), [27](http://www.informatics.jax.org/marker/MGI:1922300) | - [Klp31E](http://flybase.org/reports/FBgn0032243.html)/ Klp31D | all stages; strong in **NS** | Kif21A, 21B |
|  |  |  | - [Klp3A](http://flybase.org/reports/FBgn0011606.html) | all stages; moderate in **NS** | Kif4 |
| **5**  N-Kifs | 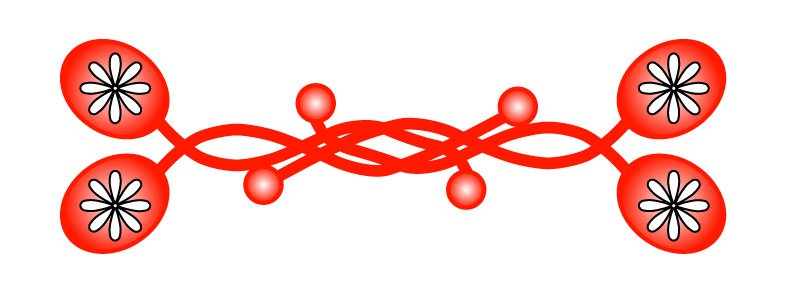 | **1** - [Kif11](http://www.informatics.jax.org/marker/MGI:1098231) | - [Klp61F](http://flybase.org/reports/FBgn0004378.html) | strong at all stages; not in **NS (?)** | Kif11 |
| **6**  N-Kifs | 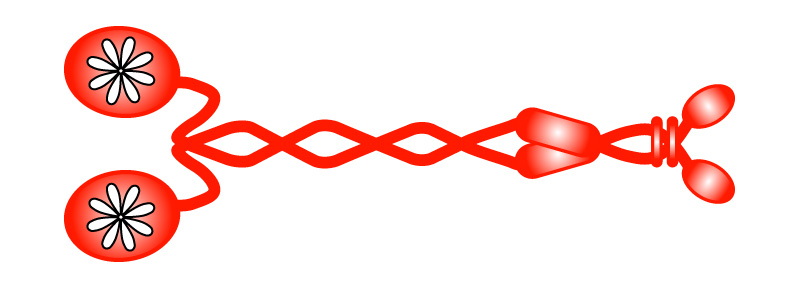 | **3** - [Kif20A](http://www.informatics.jax.org/marker/MGI:1201682), [20B](http://www.informatics.jax.org/marker/MGI:2444576), [23](http://www.informatics.jax.org/marker/MGI:1919069) | - [pavarotti](http://flybase.org/reports/FBgn0011692.html)/ pav | all stages; fat body, ovary, testes **^1^** | Kif23 |
| **12**  N-Kifs | homodimer **^2^** | **2** - [Kif12](http://www.informatics.jax.org/marker/MGI:1098232), [15](http://www.informatics.jax.org/marker/MGI:1098258) | - [Klp54D](http://flybase.org/reports/FBgn0263076.html) | embryo, late larva and pupa; high in **NS** | Kif12 |
| **13**  M-Kifs | 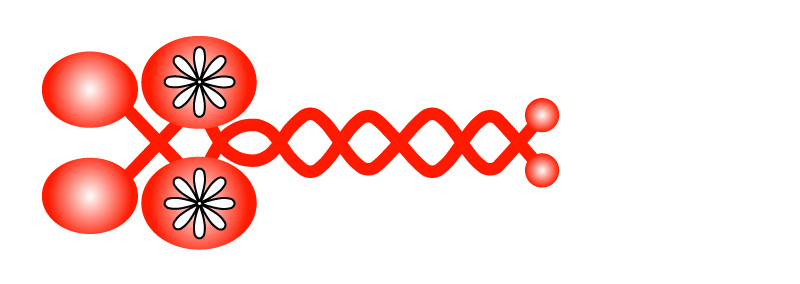 | **4** - [Kif2A](http://www.informatics.jax.org/marker/MGI:108390), [2B](http://www.informatics.jax.org/marker/MGI:1920720), [2C](http://www.informatics.jax.org/marker/MGI:1921054), [24](http://www.informatics.jax.org/marker/MGI:1918345) | - [Klp10A](http://flybase.org/reports/FBgn0030268.html) | all stages; strong in **NS** | Kif2A, 2B, 2C |
|  |  |  | - [Klp59C](http://flybase.org/reports/FBgn0034824.html) | pupal stage; in testis |  |
|  |  |  | - [Klp59D](http://flybase.org/reports/FBgn0034827.html) | pupal stage; testis, fat body |  |

| **Type** | **Structure** | **All**  **in mouse** | **Fly genes** | **Expression of fly kinesins** | **Assigned mouse homolog** |
| --- | --- | --- | --- | --- | --- |
| HC | 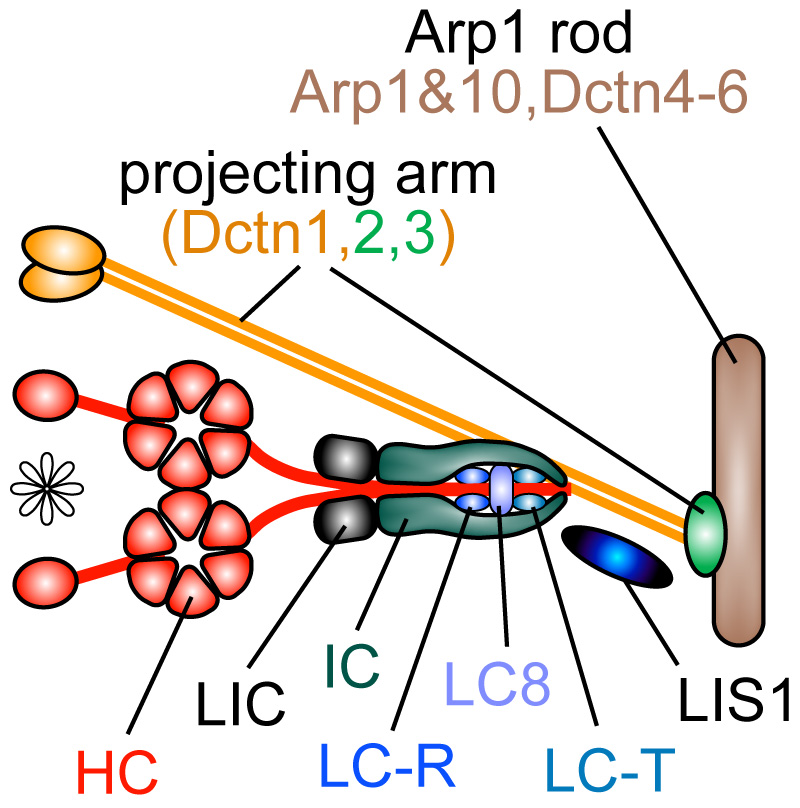 | **2 -** [Dync1h1](http://www.informatics.jax.org/marker/MGI:103147), [2h1](http://www.informatics.jax.org/marker/MGI:107736) | - [Dhc64C](http://flybase.org/cgi-bin/fbidq.html?FBgn0261797) | all stages, strong in **NS** | Dync1h1, 2h1 |
|  |  |  | - [btv](http://flybase.org/cgi-bin/fbidq.html?FBgn0023096)/Dhc36D | all stages, strong in **NS** |  |
| LC8 |  | **2 -** [Dynll1](http://www.informatics.jax.org/marker/MGI:1861457), [2](http://www.informatics.jax.org/marker/MGI:1915347) | - [ctp](http://flybase.org/reports/FBgn0011760.html)/cut up | all stages; strong in **NS** | Dynll1, 2 |
|  |  |  | - [Cdlc2](http://flybase.org/cgi-bin/fbidq.html?FBgn0026141) | adult; fat body and testis |  |
| LC-T |  | **5 -** [Dynlt1a](http://www.informatics.jax.org/marker/MGI:3807506), [1b](http://www.informatics.jax.org/marker/MGI:98643), [1c](http://www.informatics.jax.org/marker/MGI:3807476), [1f](http://www.informatics.jax.org/marker/MGI:3780996), [3](http://www.informatics.jax.org/marker/MGI:1914367) | - [Dlc90F](http://flybase.org/cgi-bin/fbidq.html?FBgn0024432) | all stages, strong in **NS** | Dynlt1, 3 |
| LC-R |  | **2 -** [Dynlrb1](http://www.informatics.jax.org/marker/MGI:1914318), [2](http://www.informatics.jax.org/marker/MGI:1922715) | - [robl](http://flybase.org/cgi-bin/fbidq.html?FBgn0024196)/ roadblock | all stages; strong in **NS** | Dynlrb1, 2 |
|  |  |  | - [robl22E](http://flybase.org/cgi-bin/fbidq.html?FBgn0028570) **^3^** | adult; fat body and testis |  |
|  |  |  | - [CG10834](http://flybase.org/cgi-bin/fbidq.html?FBgn0032972) | adult; fat body and testis |  |
| IC |  | **2 -** [Dync1i1](http://www.informatics.jax.org/marker/MGI:107743), [2](http://www.informatics.jax.org/marker/MGI:107750) | - [sw](http://flybase.org/cgi-bin/fbidq.html?FBgn0003654)/short wing | all stages; NS? | Dync1i1, 2 |
| LIC |  | **2 -** [Dync1li1](http://www.informatics.jax.org/marker/MGI:2135610), [2](http://www.informatics.jax.org/marker/MGI:107738) | - [Dlic](http://flybase.org/cgi-bin/fbidq.html?FBgn0030276) **^4^** | all stages; strong in **NS** | Dync1li1, 2 |
|  |  |  | - [CG3769](http://flybase.org/cgi-bin/fbidq.html?FBgn0032119) | all stages; mild in **NS** | Dync2li1 |
| dyn-actin and other |  | **1** - [Dctn1](http://www.informatics.jax.org/marker/MGI:107745)/p150 | - [Gl](http://flybase.org/cgi-bin/fbidq.html?FBgn0001108)/Glued | all stages, strong in **NS** | Dctn1 |
|  |  |  | - [CG9279](http://flybase.org/cgi-bin/fbidq.html?FBgn0036882) | all stages; also NS |  |
|  |  | **1** - [Dctn2](http://www.informatics.jax.org/marker/MGI:107733)/p50 | - [Dmn](http://flybase.org/cgi-bin/fbidq.html?FBgn0021825) | all stages, strong in **NS** | Dctn2 |
|  |  | **1** - [Dctn3](http://www.informatics.jax.org/marker/MGI:1859251)/p24 | - [l(2)06496](http://flybase.org/reports/FBgn0010622.html) | all stages, strong in **NS** | Dctn3 **^5^** |
|  |  | **1** - [Dctn4](http://www.informatics.jax.org/marker/MGI:1914915)/p62 | - [CG12042](http://flybase.org/cgi-bin/fbidq.html?FBgn0033206) | all stages; strong in **NS** | Dctn4 |
|  |  | **1** - [Dctn5](http://www.informatics.jax.org/marker/MGI:1891689)/p25 | - [dyn-p25](http://flybase.org/cgi-bin/fbidq.html?FBgn0040228) | all stages, strong in **NS** | Dctn5 |
|  |  | **1** - [Dctn6](http://www.informatics.jax.org/marker/MGI:1343154)/p27 | - [l(2)37Ce](http://flybase.org/cgi-bin/fbidq.html?FBgn0086446) | all stages, strong in **NS** | Dctn6 |
|  |  | **2** - [Actr1a](http://www.informatics.jax.org/marker/MGI:1858964), [1b](http://www.informatics.jax.org/marker/MGI:1917446) | - [Arp1](http://flybase.org/reports/FBgn0011745.html) | all stages, strong in **NS** | Actr1a, 1b |
|  |  | **1 -** [Actr10](http://www.informatics.jax.org/marker/MGI:1891654) | - [Arp10](http://flybase.org/reports/FBgn0031050.html)/ ARP11 | all stages, strong in **NS** | Actr10 |
|  |  | **1 -** [Pafah1b1](http://www.informatics.jax.org/marker/MGI:109520)/ Lis1 | - [Lis-1](http://flybase.org/cgi-bin/fbidq.html?FBgn0015754) | all stages, strong in **NS** | Pafah1b1 |

Additional file 1: **Table S1. Microtubule** **(MT)-associated motor proteins with roles in axons.**

**Column 1**: the classification of kinesin types (N-KIFs, N-terminal motor domain KIFs; M-KIFs, middle motor domain KIFs) and of subunits of the dynein/dynactin complex (HC, heavy chain; LC8, type 8 light chain; LC-R, roadblock-type light chain; LC-T, Tctex-type light chain); **column 2**: diagram of respective motors modified from [61, 98-101] (motor domains indicated by asterisks); **column 3**: the reported mouse genes for each motor protein or complex component according to [34], NCBI gene resource and Mouse Genome Informatics (MGI), hyperlinked to MGI; **column 4**: the *Drosophila* genes encoding the respective motor proteins or complex components, hyper-linked to their entries on FlyBase [102]; **column 5**: expression timeline and potential nervous system (NS) expression of the fly genes, according to G Browse in FlyBase; **column 6:** mouse homologues of the fly genes, as listed in FlyBase. Comments: (1) but early neuronal phenotypes were reported [103]; (2) according to [62]; (3) for additional listings (robl37BC, robl62A, robls54B) no non-Drosophilid homologues are given; (4) for the additional listing Dic61B no non-Arthropod Metazoa orthologues are given; (5) according to [104]. Information in square brackets is hyperlinked to ENSEMBL entries: CAP-Gly, EB1-binding domain; PH, membrane-binding pPlekstrin hHomology domain; PX, membrane-binding Phox domain.
