# Supplementary material for: The intricate relationship between microtubules and their associated motor proteins during axon growth and maintenance
Source: Neural Dev. 2013 Sep 8;8:17. doi: 10.1186/1749-8104-8-17 (PMC3846809; doi:10.1186/1749-8104-8-17)
Supplement: Additional file 2 — Regulation of axonal transport [10,12,19,34,36,71,97,98,105,106,107,108,109,110,111,112,113,114,115,116,117,118]. [file 1749-8104-8-17-S2.doc]

Additional file 2 Regulation of axonal transport.

Cargo transport occurs in intervals. It stops and reverts constantly, and the overall direction of cargo movement is the net function of these events. The underlying mechanisms include occasional back-stepping of motors, cargo swap between kinesins or dyneins, switches between active or inactive states of motors; and motors can even be transported passively with cargo or diffuse along MTs when in a folded or inhibited state [105]. The correct balance between these various states is the product of a complex regulatory network.

  ▪ as discussed elsewhere [34,36,71], the fundamental principal underpinning specific cargo transport is that different classes of motor proteins (Additional file 1: Table S1) have context-dependent preferences for different cargos and for distinct cargo linkers. Recently published examples of the small G protein arl-8 or the scaffolding protein JIP1 (JNK-interacting protein 1) nicely demonstrate how linkers can regulate transport at the level of cargo availability, motor-specific linkage and even motor activation [106,107].

  ▪ signaling pathways provide important switches in this context, changing the phosphorylation state of motors, cargo linkers and other associated proteins to determine their activation state as well as cargo transport direction [105]. Further posttranslational modifications on cargo, such as palmitoylation have been reported [98]

  ▪ structural features of cells regulate transport, such as the organization of MTs into unipolar parallel bundles in axons versus anti-parallel bipolar bundles in dendrites, or the selective barrier function of the uniquely structured axon initiation zone [105]. Also the distinctly organized F-actin networks in axons and dendrites [108] are likely to add to this scenario.

  ▪ specific posttranslational modifications on MTs (Figure 3) are believed to prime or block MTs for transport, either directly or through interaction with other MTBPs [10-12], and the higher content of GTP-tubulin typically found in axonal MTs is important for kinesin (KIF5) processivity [19].

  ▪ motors can co-operate. For example, several dyneins can join up and work as a team to generate larger forces and maintain velocity when transporting high load cargos [36,109]. Furthermore, there is an intricate and little understood functional relationship between kinesins and dyneins which display mutual dependence during transport in certain contexts [36,110,111].

  ▪ there is intense cross-talk of MT motors with other classes of MTBPs. For example, doublecortin regulates MT plus ends in neuronal growth cones [112], but it also binds the motor domains of Kif1A and enhances its MT association [97]. MAP7/ensconsin physically interacts with kinesin-1 and promotes its function [113,114]. Binding of tau to MTs was long believed to inhibit axonal transport [115], but has also been shown to promote kinesin-1 transport in a tau isoform-specific manner, especially when bound to GTP-rich MTs [116]. In some neurodegenerative diseases, filamentous tau is prevalent and can trigger signaling pathways causing cargo release from conventional kinesins, that is, inhibit kinesin-dependent transport [117]. Vice versa, reduced kinesin-1-mediated transport enhances tau hyperphosphorylation known to trigger the formation of filamentous tau, thus potentially establishing a vicious circle [118].
