# Supplementary material for: The intricate relationship between microtubules and their associated motor proteins during axon growth and maintenance
Source: Neural Dev. 2013 Sep 8;8:17. doi: 10.1186/1749-8104-8-17 (PMC3846809; doi:10.1186/1749-8104-8-17)
Supplement: Additional file 3 — Using the fruit fly Drosophila to study the cytoskeleton during axon growth [23,85,89,91,119,120,121,122]. [file 1749-8104-8-17-S3.doc]

Additional file 3: Using the fruit fly *Drosophila* to study the cytoskeleton during axon growth.

Together with the nematode *Caenorhabditis elegans*, *Drosophila* is certainly the best established invertebrate genetic model organism [119]. For a number of reasons the fly is in a good position to contribute conceptual understanding to work on the neuronal cytoskeleton:

• flies are cheap to keep, fast developing and experimentally amenable, and work in the fly nervous system has an impressive track-record in delivering fundamental and evolutionary conserved understanding [89];

• their cytoskeleton-associated genes including motors (Additional file 1: Table S1) are well-conserved, and loss-of-function of homologous genes causes comparable phenotypes in neurons of fly and vertebrates/mammals [85,91]

• cytoskeleton-associated genes of the fly displays relatively low genomic redundancy (Add 1 Table 1), they are highly amenable to genetic, molecular and transgenic manipulations, and genetic tools tend to be readily available [23,120];

• the study of cytoskeletal machinery in axons can make use of excellent functional readouts both *in vivo* and in culture, and the cytoskeletal dynamics in fly neurons described so far are highly reminiscent of those in vertebrate neurons [121,122];

• finally, *Drosophila* research benefits from an open and well organized community of fly researchers and is easy to establish even in laboratories without fly experience [119]
